# SARS-CoV-2 vaccine response and rate of breakthrough infection in patients with hematological disorders

**DOI:** 10.1186/s13045-022-01275-7

**Published:** 2022-05-07

**Authors:** José Luis Piñana, Lucia López-Corral, Rodrigo Martino, Lourdes Vazquez, Ariadna Pérez, Gabriel Martin-Martin, Beatriz Gago, Gabriela Sanz-Linares, Andrés Sanchez-Salinas, Lucia Villalon, Venancio Conesa-Garcia, María T. Olave, Magdalena Corona, Sara Marcos-Corrales, Mar Tormo, José Ángel Hernández-Rivas, Juan Montoro, Alicia Rodriguez-Fernandez, Irene Risco-Gálvez, Pablo Rodríguez-Belenguer, Juan Carlos Hernandez-Boluda, Irene García-Cadenas, Montserrat Ruiz-García, Juan Luis Muñoz-Bellido, Carlos Solano, Ángel Cedillo, Anna Sureda, David Navarro, the Infectious Complications Subcommittee of the Spanish Hematopoietic Stem Cell Transplantation and Cell Therapy Group (GETH-TC)

**Affiliations:** 1 Division of Clinical Hematology, Hematology Department, Hospital Clínico Universitario de Valencia, Avda Blasco Ibañez, 17, 46010 Valencia, Spain; 2 Fundación INCLIVA, Instituto de Investigación Sanitaria Hospital Clínico Universitario de Valencia, Valencia, Spain; 3 Hematology Division, Hospital Universitario de Salamanca, Salamanca, Spain; 4 Hematology Division, Hospital de la Santa Creu i Sant Pau, Barcelona, Spain; 5 Hematology Division, Hospital Regional Universitario Carlos Haya, Malaga, Spain; 6 Hematology Division, Institut Català Oncologia-Hospital Duran i Reynals, IDIBELL, Universitat de Barcelona, Barcelona, Spain; 7 Hematology Division, Hospital Clínico Universitario Virgen de la Arrixaca, Murcia, Spain; 8 Hematology Division, Hospital Universitario Fundación Alcorcón, Madrid, Spain; 9 Hematology Division, Hospital General Universitario de Elche, Elche, Spain; 10 Hematology Division, Hospital Clínico Universitario Lozano Blesa, IIS Aragon, Saragossa, Spain; 11 Hematology Division, Hospital Ramon y Cajal, Madrid, Spain; 12 Hematology Division, Hospital Universitario Infanta Leonor, Madrid, Spain; 13 Hematology Division, Hospital universitario y politécnico La Fe, Valencia, Spain; 14 Hematology Division, Hospital Universitario Virgen Macarena, Seville, Spain; 15 Hematology Division, Hospital Arnau de Vilanova, Valencia, Spain; 16 Research Program on Biomedical Informatics (GRIB), Department of Experimental and Health Sciences, Hospital del Mar Medical Research Institute (IMIM), Universitat Pompeu Fabra, Barcelona, Spain; 17 Hospital General Universitari d’Elx, Elche, Spain; 18 Hospital Universitario de Salamanca, Salamanca, Spain; 19 Department of Medicine, School of Medicine, University of Valencia, Valencia, Spain; 20 Hematopoietic Stem Cell Transplantation and Cell Therapy Group (GETH) Office, Madrid, Spain; 21 Hospital Clinico Universitario de Valencia, Valencia, Spain

**Keywords:** SARS-CoV-2 vaccines, Breakthrough SARS-CoV-2 infection, Correlates of protection, Hematological malignancies, Allogeneic stem cell transplantation, Autologous stem cell transplantation, COVID-19, Vaccine, Immunocompromised patients, Moderna mRNA-1273, Pfizer-BioNTech BNT162b2

## Abstract

**Background:**

The clinical efficacy of SARS-CoV-2 vaccines according to antibody response in immunosuppressed patients such as hematological patients has not yet been established.

**Patients and methods:**

A prospective multicenter registry-based cohort study conducted from December 2020 to December 2021 by the Spanish transplant and cell therapy group was used to analyze the relationship of antibody response at 3–6 weeks after full vaccination (2 doses) with breakthrough SARS-CoV-2 infection in 1394 patients with hematological disorders.

**Results:**

At a median follow-up of 165 days after complete immunization, 37 out of 1394 (2.6%) developed breakthrough SARS-CoV-2 infection at median of 77 days (range 7–195) after full vaccination. The incidence rate was 6.39 per 100 persons-year. Most patients were asymptomatic (19/37, 51.4%), whereas only 19% developed pneumonia. The mortality rate was 8%. Lack of detectable antibodies at 3–6 weeks after full vaccination was the only variable associated with breakthrough infection in multivariate logistic regression analysis (Odds Ratio 2.35, 95% confidence interval 1.2–4.6, *p* = 0.012). Median antibody titers were lower in cases than in non-cases [1.83 binding antibody units (BAU)/mL (range 0–4854.93) vs 730.81 BAU/mL (range 0–56,800), respectively (*p* = 0.007)]. We identified 250 BAU/mL as a cutoff above which incidence and severity of the infection were significantly lower.

**Conclusions:**

Our study highlights the benefit of developing an antibody response in these highly immunosuppressed patients. Level of antibody titers at 3 to 6 weeks after 2-dose vaccination links with protection against both breakthrough infection and severe disease for non-Omicron SARS-CoV-2 variants.

**Supplementary Information:**

The online version contains supplementary material available at 10.1186/s13045-022-01275-7.

## Introduction

The coronavirus infectious disease 2019 (COVID-19) pandemic caused by the new coronavirus (SARS-CoV-2) can have a dreadful impact in hematological patients, with mortality rates exceeding 25% [1–6]. SARS-CoV-2 vaccination is expected to reduce the severity of COVID-19 in these immunocompromised patients [7–11]. Although the antibody response after full SARS-CoV-2 vaccination in hematological patients is of a lower magnitude than in the general population [12–18], a clinical benefit is still expected, as is the case with influenza vaccination in allogeneic hematopoietic stem cell transplant (allo-HSCT) recipients [19]. Since no prospective randomized SARS-CoV-2 vaccine trials have been conducted in these patients, vaccine efficacy data are lacking in this scenario.

The current study analyzes the clinical benefit of full SARS-CoV-2 vaccination (2 doses) through an observational registry conducted by the Spanish Hematopoietic Stem Cell Transplantation and Cell Therapy Group (GETH-TC) aimed at monitoring the response to SARS-CoV-2 vaccine and the breakthrough SARS-CoV-2 infections over time in a large cohort of 1394 patients with hematological disorders.

## Patients and methods

### Study population

This is a prospective multicenter registry-based cohort study conducted by the Infectious Complications Subcommittee (GRUCINI) of the GETH-TC in collaboration with the Spanish Society of Hematology and Hemotherapy (SEHH). Details of this registry have been previously described elsewhere [20, 21]. In brief, the registry included consecutive adult patients with a prior history of hematological disorders who were fully vaccinated against SARS-CoV-2 between December 30, 2020, and June 30, 2021, in 21 participating Spanish centers. Patients were followed and monitored for development of SARS-CoV-2-reactive IgG antibodies (SCoV2-R-A) and breakthrough SARS-CoV-2 infection at different time points (3–6 weeks, 3, 6 and 12 months) after the complete vaccination schedule (defined as two vaccine doses). The status of all included patients was updated on December 1, 2021. All patients included in this registry gave their signed informed consent in accordance with the declaration of Helsinki. The local research ethical committee of the Hospital Clínico Universitario of Valencia approved the registry and study protocol (reference code 35.21).

### Inclusion criteria and cohort selection

As of December 1, 2021, the GETH-TC registry included 1683 patients with different hematological disorders who had been fully vaccinated against COVID-19. With the aim of assessing the risk of breakthrough SARS-CoV-2 infection according to antibody detection at 3–6 weeks after the second vaccine dose, the current study focused on patients with available serological testing 3 to 6 weeks after the second vaccine dose. We excluded 289 patients from 3 centers, initially included with limited data (only filiation data), that did not obtain the institutional approval for serological testing and 1394 hematological patients were included in the final study analysis.

### Technical considerations and definitions

Antibody detection or seropositivity was defined when SARS-CoV-2-reactive IgG antibodies (SCoV2-R-A) were detected at any level above the lower limit of detection level for each tests used. We assessed seropositivity using serological ELISA or chemiluminescence immunoassay following manufacturer instructions according to their availability at the microbiology services of each participating center. As recommended by the SEHH, in vaccinated individuals serological testing included the detection of IgG against both the nucleocapsid (N) and surface (S) proteins (anti-N and anti-S IgG, respectively) [7]. Of the 1394 patients included, 1244 (89%) received quantitative assessment, whereas the remainder was assessed through qualitative testing. Antibody levels were normalized according to the WHO standard, and results were reported as SCoV2-R-A binding antibody units per milliliter (BAU/mL). Additional file 1: Table S1 summarizes the technical characteristics of the serological tests used and normalization of antibody titers to BAU/mL according to WHO standards.

Pre-vaccination SARS-CoV-2 infection was defined as patients with prior history of PCR-proven COVID-19 and/or positive SARS-CoV-2 serostatus (IgG and/or IgM) before the first vaccine dose.

Patients with respiratory symptoms underwent PCR screening for the development of SARS-CoV-2 according to the treating physician criteria. Breakthrough SARS-CoV-2 infection was defined as molecular (PCR test) or humoral (anti-N seroconversion between two consecutive serological tests) evidence of SARS-CoV-2 infection 7 days after the second vaccine dose until last follow-up. The PCR tests used are provided in Additional file 1: Table S2.

### Endpoints and statistical analysis

The primary objective of the study was to assess the occurrence of breakthrough SARS-CoV-2 infection and its correlation with qualitative and quantitative humoral response at 3–6 weeks after full COVID-19 vaccination. We also analyzed the effect of different cutoff values of quantitative SCoV2-R-A titers on development and severity of breakthrough SARS-CoV-2 infection.

The main patient characteristics were reported by descriptive statistics on the total available information: Medians and ranges were used for continuous variables, while absolute and percentage frequencies were used for categorical variables. For comparisons, Fisher exact test or Mann–Whitney’s *U* test was used when appropriate. Univariate and multivariate analyses were tested using logistic regression models. Variables with a *p* value ≤ 0.1 in the univariate model were included in the multivariate analysis. A *p* value < 0.05 was considered statistically significant. All *p* values are two-sided. A median test sub-analysis to check the protective effect of the amount of SCoV2-R-A was carried out in patients with available quantitative SCoV2-R-A titers normalized to BAU/mL. All analyses were performed using the statistical software SPSS v. 25(IBM SPSS Statistics, Armonk, New York, USA).

## Results

### Patient characteristics

Patient characteristics are summarized in Table 1. Most patients (*n* = 1345, 96.5%) received complete vaccination with mRNA vaccines, and their median age was 63 years (range 18–97). Overall, the most common hematological diseases were B-cell non-Hodgkin’s lymphoma (B-cell NHL) (*n* = 302, 21.6%), plasma cell disorders (PCD) (*n* = 236, 16.9%), acute myeloid leukemia (AML) (*n* = 179, 12.8%), chronic lymphocytic leukemia (CLL) (*n* = 158, 11.3%) and chronic myeloproliferative neoplasms (cMPN) (*n* = 139, 10%). Among the cell therapy procedures, the most common was allo-HSCT (*n* = 369, 26.5%) followed by autologous stem cell transplantation (*n* = 110, 8%) and chimeric antigen receptor of T cell (CAR-T) therapy (*n* = 21, 1.5%). Note that this series included 109 patients (8.4%) with prior PCR and/or serological proof of SARS-CoV-2 infection before being vaccinated. Median follow-up after the second vaccine dose was 165 days (range 12–269).

**Table 1:** Patient characteristics

Characteristics	(*n* = 1394)
Prior COVID-19, *n* (%)	109 (7.9)
Diagnosed by PCR	95 (7)
Positive serostatus prior to vaccination	37 (2.6)
Negative serostatus prior to vaccination	13 (1)
Detected by pre-vaccine serological test	14 (1.5)
Median time from COVID-19 to vaccination, days (range)	185 (33–460)
Serological status prior to vaccination, *n* (%)	
Positive	50 (4)
Negative	422 (30)
Not tested	922 (66)
Median time from serology to vaccination, days (range)	0 (0–386)
Type of vaccine, *n* (%)	
Moderna mRNA-1273	983 (70.5)
Pfizer-BioNTech BNT162b2	362 (26)
Adenoviral vector-based	49 (3.5)
Age (years), median (range)	63 (18–97)
18–40 years, *n* (%)	143 (10)
41–60 years, *n* (%)	496 (35.5)
61–70 years, *n* (%)	373 (26.8)
> 71 years, *n* (%)	382 (27.4)
Male, *n* (%)	784 (56.3)
ECOG 0–1 at vaccination	1351 (97)
Baseline disease, *n* (%)	
AML	179 (12.8)
ALL	46 (3.3)
MDS	158 (11.3)
B-cell NHL	302 (21.6)
T cell NHL	38 (2.7)
Plasma cell disorders	236 (16.9)
CLL	158 (11.3)
HD	103 (7.4)
cMPN	139 (10)
Aplastic anemia	16 (1)
Non-malignant disorders	18 (1.3)
Type of cell therapy	
Allo-HSCT	369 (26.5)
ASCT	110 (8)
CAR-T	21 (1.5)
Status disease at vaccination, *n* (%)	
Complete remission	824 (59.2)
Partial remission	162 (11.6)
Active disease	408 (29.2)
Time last treatment to COVID-19 vaccine, months (range)	
Untreated	172 (12.3)
Active treatment	509 (36.5)
≥ 6 month to 1 year	92 (6.6)
≥ 1 year	621 (44.5)
Immunosuppressant drugs at vaccination, *n* (%)	300 (21.5)
Corticosteroids at vaccination, *n* (%)	255 (18.6)
Daratumumab, *n* (%)	46 (3.3)
Venetoclax, *n* (%)	14 (1)
Anti-CD-20 moAb, *n* (%)	241 (17.3)
< 6 months before 1st vaccine dose	87 (6.2)
6 to 1 year before 1st vaccine dose	25 (1.8)
> 1 year before 1st vaccine dose	129 (9.3)
BTK inhibitor therapy, *n* (%)	63 (4.5)
TKI therapy, *n* (%)	40 (2.9)
Lenalidomide maintenance, *n* (%)	120 (8.6)
Ruxolitinib therapy, *n* (%)	14 (1)
Blood count before vaccination (× 10^9^/mL)	
Absolute neutrophile counts, median (range)	3.1 (0–46.7)
Absolute lymphocyte counts, median (range)	1.73 (0.14–262.1)
Absolute lymphocyte counts < 1 × 10^9^/L	265 (18.6)
Time from 2nd dose to first serologies, median days (range)	21 (12–62)
Median time between vaccine doses, median days (range)	28 (17–115)
SCoV2-R-A detection at 3–6 weeks after full vaccination, *n* (%)	1090 (78.2)
Patient with SCoV2-R-A titers at 3–6 weeks in BAU/mL, *n* (%)	1244 (89%)
Median SCoV2-R-A titers at 3–6 weeks in BAU/mL, (range)	715 (0–56,800)
Third vaccine dose given, *n* (%)	550 (39.5)
Time from 2nd dose to 3rd dose, days (range)	153 (39–269)
Median follow-up after full vaccination, days (range)	165 (12–269)
COVID-19 after vaccination, *n* (%)	37 (2.7)
Median time from vaccination to SARS-CoV-2 infection, days (range)	77 (7–195)

PCR, Polymerase chain reaction AML, acute myeloid leukemia; ALL, acute lymphoblastic leukemia; MDS, myelodysplastic syndrome; B-cell NHL, B-cell non-Hodgkin lymphoma; T cell NHL, T cell non-Hodgkin lymphoma; CLL, chronic lymphocytic leukemia; HD, Hodgkin disease; MPN, chronic myeloproliferative neoplasm; Allo-HSCT, allogeneic stem cell transplantation; ASCT, autologous stem cell transplantation; CAR-T, T cell chimeric antigen receptor; moAb, monoclonal antibody; BTK inhibitor, Bruton’s tyrosine kinase inhibitor; TKIs, tyrosine kinase inhibitors; and SCoV2-R-A, SARS-CoV-2-reactive IgG antibodies

**Fig. 1:**
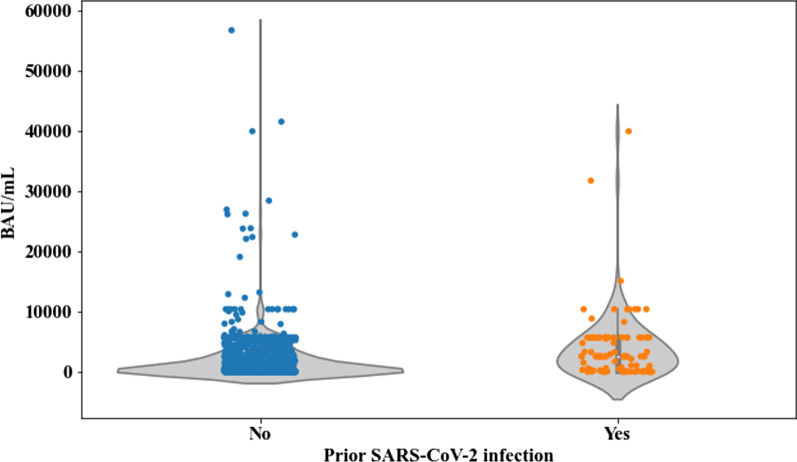
Median anti-SARS-CoV-2 IgG-reactive antibodies titers measured in binding antibody units/mL (BAU/mL) at 3–6 weeks after the 2nd dose according to pre-vaccination SARS-CoV-2 infection. Patients with SARS-CoV-2 infection prior to vaccination (*n* = 109) had a median of 2550 BAU/mL (range 0–10,400) vs those without prior history of SARS-CoV-2 infection (*n* = 1118) median 493.6 BAU/mL (range 0–6338.6) (*p* < 0.0001)

Overall, the SCoV2-R-A detection rate at 3–6 weeks after the complete vaccination was 78.2%. Among those with quantitative antibody testing, the median SCoV2-R-A titer was 720.26 BAU/mL (range 0–58,600). We compared SCoV2-R-A titers at 3–6 weeks after full vaccination in patients with and without SARS-CoV-2 infection prior to vaccination (excluding 7 patients with breakthrough SARS-CoV-2 infection after the second vaccine dose and before the first serological testing) and found higher titers in those with (median 2550 BAU/mL, range 0–10,400) vs those without (median 493.6 BAU/mL, range 0–6338.6) (*p* < 0.0001) infection (Fig. 1).

### Breakthrough SARS-CoV-2 infection

We identified 37 patients (2.6%) with breakthrough SARS-CoV-2 infection at median of 77 days (range 7–195) after the second vaccine dose. The overall incidence rate of breakthrough SARS-CoV-2 infection was 6.39 per 100 persons-year. The main clinical and SARS-CoV-2 breakthrough infection characteristics are detailed in Table 2. Most patients were diagnosed through molecular PCR testing (*n* = 22, 60%), whereas those remaining (*n* = 15, 40%) were diagnosed by seroconversion of anti-N between two consecutive serological tests. Seven patients (19%) developed SARS-CoV-2 infection between the second vaccine dose and SCoV2-R-A testing at 3–6 weeks, with an incidence rate of 6.14 per 100 persons-year. Twelve patients (32%) developed the infection between 3–6 weeks and 3 months after the complete vaccination with an estimated incidence rate of 6.9 per 100 persons-year. Finally, 18 patients had the infection between 3 and 7 months after the complete vaccination with an estimated incidence rate of 14.09 per 100 persons-year. Eighteen patients had COVID-19 (48.6%), whereas 19 (51.4%) were asymptomatic. SARS-CoV-2 was detected by PCR in 4 asymptomatic patients during screening performed before hospital admission for scheduled procedures/treatments. Pneumonia was documented in 7 cases (19%), whereas the SARS-CoV-2 infection-related hospital admission rate was 32% (*n* = 12). There were 3 COVID-19-related deaths (8%) at a median of 26 days (range 7–67) after SARS-CoV-2 detection.

**Table 2:** Characteristics of patients with breakthrough SARS-CoV-2 infection

Characteristics	SARS-CoV-2 infection (*n* = 37)
Prior COVID-19, n /n evaluable (%)	0/109
Type of vaccine, n/n evaluable (%)	
Moderna mRNA-1273	24/982 (2.4)
Pfizer-BioNTech BNT162b2	11/362 (3)
Adenoviral vector-based	2/50 (4)
Age (years), n/n evaluable (%)	
18–40 years	6/144 (4.2)
41–60 years	17/495 (3.4)
61–70 years	6/373 (1.6)
> 71 years	8/382 (2)
Male, n (%)/n evaluable (%)	25/784 (3.2)
Baseline disease, n/n evaluable (%)	
AML	5/180 (2.7)
ALL	1/46 (2.1)
MDS	5/158 (3.1)
B-cell NHL	5/301 (1.7)
T cell NHL	3/38 (8)
Plasma cell disorders	5/236 (2.1)
CLL	4/158 (2.5)
HD	6/103 (5.8)
cMPN	2/139 (1.4)
Aplastic anemia	0/16
Non-malignant disorders	1/17 (5.5)
Cell therapy, n /n evaluable (%)	18/501 (3.6)
Type of cell therapy, n /n evaluable (%)	
Allo-HSCT	13/370 (3.5)
ASCT	5/110 (4.7)
CAR-T	0/21
Status disease at vaccination, n /n evaluable (%)	
Complete remission	21/825 (2.5)
Partial remission	6/162 (3.7)
Active disease	10/407 (2.4)
Time last treatment to COVID-19 vaccine, n /n evaluable (%)	
Untreated	7/172 (4)
Active treatment	10/509 (1.9)
≥ 6 month to 1 year	5/92 (5.4)
≥ 1 year	15/620 (2.4)
Immunosuppressant drugs at vaccination, n /n evaluable (%)	13/300 (4.3)
Corticosteroids at vaccination, n /n evaluable (%)	8/255 (3.1)
Daratumumab, n /n evaluable (%)	1/46 (2.1)
Venetoclax, n /n evaluable (%)	0/14
Anti-CD-20 moAb, n /n evaluable (%)	4/241 (1.6)
BTK inhibitor therapy, n /n evaluable (%)	3/63 (4.7)
TKI therapy, n /n evaluable (%)	1/40 (2.5)
Lenalidomide, n /n evaluable (%)	2/120 (1.7)
Ruxolitinib therapy, n /n evaluable (%)	0/14
Absolute lymphocyte counts < 1 × 10^9^/L, n /n evaluable (%)	9/260 (3.4)
Intervals from 2nd dose to SARS-CoV-2 infection, n /n patients at risk (%)	
At 30 days after 2nd dose	14/1361 (1)
At 60 days after 2nd dose	3/1309 (0.2)
At 90 days after 2nd dose	8/1227 (0.6)
At 180 days after 2nd dose	12/518 (2.3)
SCoV2-R-A detection at 3–6 weeks, n /n evaluable (%)	17/30 (57)
Median SCoV2-R-A titer at 3–6 weeks, BAU/mL (range) [27 evaluable patients]	1.83 (0–4854.95)
SARS-CoV-2 infection after the third vaccine dose, n (%)	2/541 (0.3)
SARS-CoV-2 diagnosis, n /n evaluable (%)	
PCR	22/37 (60)
Seroconversion of anti-N antibodies	15/37 (40)
Symptomatic SARS-CoV-2 infection, n /n evaluable (%)	18/37 (48.6)
Pneumonia, n /n evaluable (%)	7/37 (19)
Hospital admission, n /n evaluable (%)	12/37 (32)
Oxygen requirement, n /n evaluable (%)	10/37 (27)
ICU admission, n /n evaluable (%)	3/37 (8)
Death, n /n evaluable (%)	3/37 (8)
Median time to death from 2nd vaccine dose, days (range)	82 (59–100)

AML, Acute myeloid leukemia; ALL, acute lymphoblastic leukemia; MDS, myelodysplastic syndrome; B-cell NHL, B-cell non-Hodgkin lymphoma; T cell NHL, T cell non-Hodgkin lymphoma; CLL, chronic lymphocytic leukemia; HD, Hodgkin disease; MPN, chronic myeloproliferative neoplasm; Allo-HSCT, allogeneic stem cell transplantation; ASCT, autologous stem cell transplantation; CAR-T, T cell chimeric antigen receptor; moAb, monoclonal antibody; BTK inhibitor, Bruton’s tyrosine kinase inhibitor; TKIs, tyrosine kinase inhibitors; SCoV2-R-A, SARS-CoV-2-reactive IgG antibodies; Anti-N, SARS-CoV-2 nucleocapsid antibodies; and ICU, intensive care unit

### Risk factors (including antibody level titers) for breakthrough SARS-CoV-2 infection

After excluding 7 patients with breakthrough SARS-CoV-2 infection before the first serological testing, we performed a univariate and multivariate regression model to assess predictors of breakthrough SARS-CoV-2 infection (Table 3). None of the 109 patients who developed COVID-19 before vaccination developed breakthrough infection (*p* = 0.1 in univariate analysis). The only variable that was found to have an impact on the risk of breakthrough SARS-CoV-2 infection in univariate and multivariate analyses was absence of SCoV2-R-A detection at 3–6 weeks after the second vaccine dose [odds ratio (OR) 2.35 95% confidence interval (CI) 1.2–4.6, *p* = 0.012].

**Table 3:** Logistic regression univariate and multivariate analyses of factors predicting SARS-CoV-2 breakthrough infection after full vaccination

Characteristics	SARS-CoV-2 infection	*p* value	SARS-CoV-2 infection	*p* value
	Univariate OR (95% CI)		Multivariate OR (95% CI)	
Prior COVID-19	0.2 (0.02–1.2)	0.1	ns	
Type of vaccine				
Moderna mRNA-1273	1			
Pfizer-BionTech BNT162b2	0.6 (0.14–2.6)	0.5		
Adenoviral vector-based	0.75 (0.16–3.5)	0.7		
Age (years)				
18–40 years	1			
41–60 years	0.8 (0.32–2.1)	0.67		
61–70 years	0.37 (0.12–1.18)	0.09	ns	
> 71 years	0.49 (0.16–1.44)	0.19		
Male sex	1.6 (0.8–3.2)	0.166		
Baseline disease				
ALL	1			
AML	1.3 (0.14–11.2)	0.8		
MDS	1.47 (0.16–33)	0.7		
B-cell NHL	0.76 (0.08–6.6)	0.8		
T cell NHL	3.8 (0.38–38.4)	0.25		
Plasma cell disorders	0.97 (0.11–8.5)	0.9		
CLL	1.16 (0.12–10.7)	0.9		
HD	2.78 (0.32–23.8)	0.35		
cMPN	0.65 (0.05–7.4)	0.7		
Aplastic anemia	0.000	0.99		
Non-malignant disorders	2.6 (0.15–44.7)	0.5		
Status disease at vaccination				
Complete remission	1			
Partial remission	1.47 (0.58–3.7)	0.4		
Active disease	0.92 (0.45–2.06)	0.9		
Time from last treatment to COVID-19 vaccine				
Untreated	1			
Under treatment	0.47 (0.17–1.26)	0.13		
> 6 months to 1 year	1.35 (0.41–4.39)	0.6		
≥ 1 year	0.58 (0.23–1.45)	0.24		
Cell therapy				
Yes	0.58 (0.3–1.1)	0.1	ns	
No	1			
Allo-HSCT	1.6 (0.82–3.4)	0.15		
ASCT	2.19 (0.8–5.98)	0.12		
CAR-T	0.00	0.99		
Corticosteroids at vaccination	1.2 (0.56–2.7)	0.59		
Daratumumab	0.8 (0.1–6)	0.83		
Venetoclax	0.00	0.99		
Anti-CD-20 moAb	0.57 (0.2–1.6)	0.29		
Bruton’s TKI therapy	1.9 (0.57–6.4)	0.29		
TKI therapy	0.95 (0.12–7)	0.9		
Lenalidomide	0.6 (0.14–2.5)	0.48		
Ruxolitinib therapy	0.00	0.99		
SCoV2-R-A negative at 3–6 weeks after 2 doses	2.5 (1.3–4.9)	0.007	2.35 (1.2–4.6)	0.012
Lymphocyte count < 0.5 × 10^9^/L	0.75 (0.09–5.4)	0.75		
Lymphocyte count < 1.0 × 10^9^/L	1.5 (0.7–3.3)	0.27		

AL, Acute leukemia; MDS, myelodysplastic syndrome; B-cell NHL, B-cell non-Hodgkin lymphoma; MM, multiple myeloma; CLL, chronic lymphocytic leukemia; HD, Hodgkin disease; MPN, chronic myeloproliferative neoplasm; Allo-HSCT, allogeneic stem cell transplantation; ASCT, autologous stem cell transplantation; moAb, monoclonal antibody; TKIs, tyrosine kinase inhibitors; and SCoV2-R-A, SARS-CoV-2-reactive IgG antibodies

**Fig. 2:**
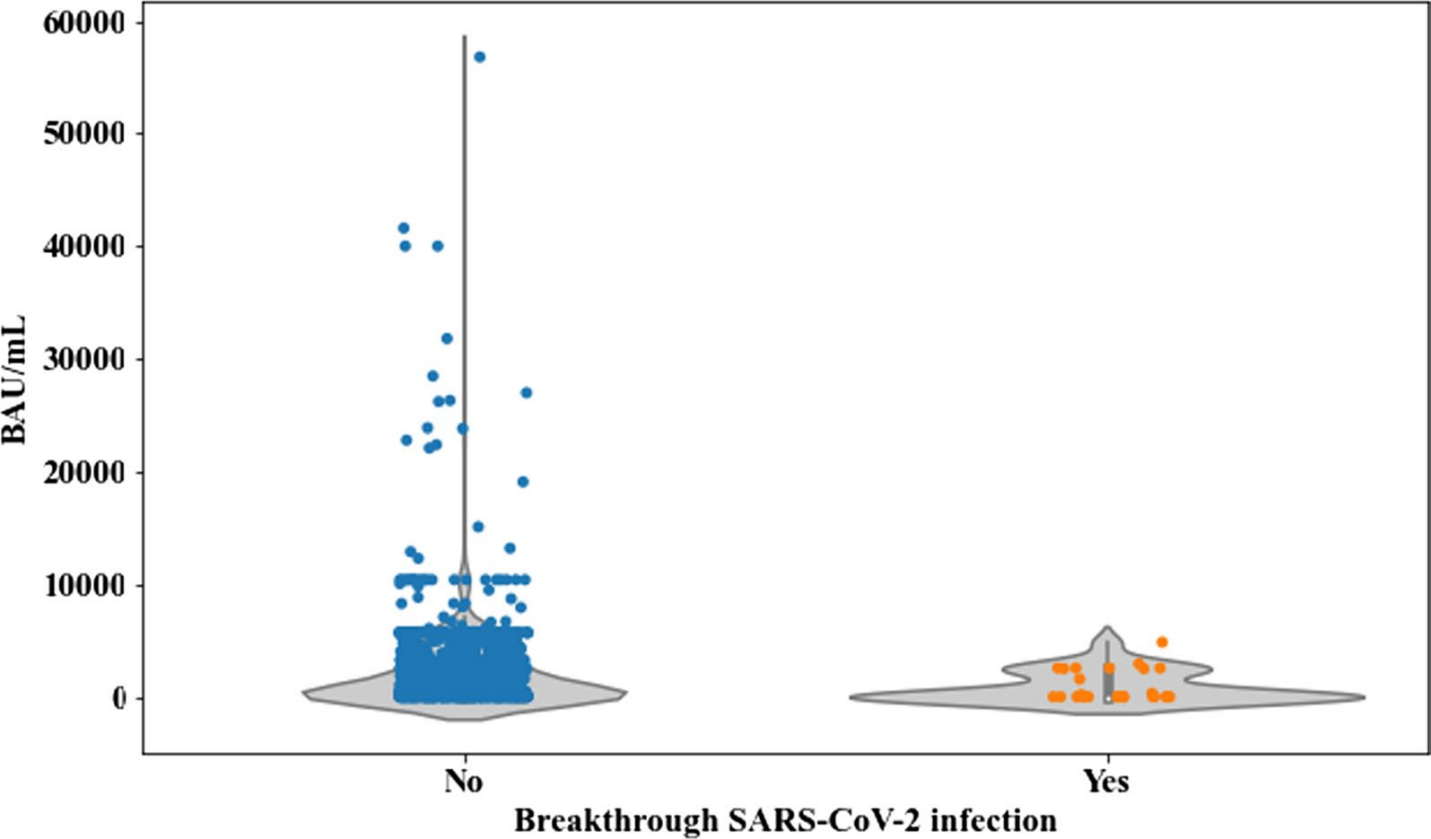
Median anti-SARS-CoV-2 IgG-reactive antibody titers measured in binding antibody units/mL (BAU/mL) at 3–6 weeks after the 2nd dose according to SARS-CoV-2 breakthrough infection. Patients who did not developed SARS-CoV-2 infection (*n* = 1207) had a median of 730.81 BAU/mL (range 0–58,600) vs 1.83 BAU/mL (range 0–4854.93) in those who did develop breakthrough infection (*n* = 27) (*p* = 0.007)

Regarding the impact of the magnitude of SCoV2-R-A load, we restricted the analysis to 27 patients, since 7 cases became infected before the first antibody determination after vaccination and another 3 cases only had qualitative SCoV2-R-A testing. Overall, 1234 patients were evaluable for antibody levels normalized as BAU/mL. The median SCoV2-R-A levels at 3–6 weeks after the full vaccination were significantly lower in the 27 patients with breakthrough SARS-CoV-2 infection as compared to those without [1.83 BAU/mL (range 0–4854.93) vs 730.81 BAU/mL (range 0–56,800), respectively (*p* = 0.007)] (Fig. 2). SCoV2-R-A levels were classified as “low” (< 250 BAU/mL) in 501 patients (41%) and as “high” (> 250 BAU/mL) in 542 (44%) or “very high” (> 4900 BAU/mL) in 191 (15%) cases.

Although the median SCoV2-R-A levels were higher in asymptomatic breakthrough SARS-CoV-2 infection (*n* = 13) than in those with COVID-19 (*n* = 14), the difference was not statistically significant [792.57 BAU/mL (range 0–2550) vs 0 BAU/mL (range 0–4854.93), *p* = 0.44]. Additionally, median SCoV2-R-A levels were lower, although not statistically significant, in those requiring hospital admission vs those who did not [0 BAU/mL (range 0–195.15) vs 309.57 BAU/mL (range 0–4854.93), respectively, *p* = 0.2]. However, breakthrough SARS-CoV-2 infection was more common in the group with “low” SCoV2-R-A levels, as was COVID-19, pneumonia, hospital admission and oxygen requirement (*p* ≤ 0.05 for all comparisons) (Table 4). Breakthrough SARS-CoV-2 infection severity did not show significant differences according to the timing after the second vaccine dose (after 2nd dose and first serological testing vs the first testing and 3rd months vs after the 3rd months).

**Table 4:** SARS-CoV-2 infection severity according to anti-SARS-CoV-2 IgG-reactive antibody cutoffs in the 27 evaluable patients

Variable	< 250 BAU/mL (*n* = 501)	250 to 4900 BAU/mL (*n* = 542)	> 4900 BAU/mL (*n* = 191)	*p* value
SARS-CoV-2 infection	17 (3.4%)	10 (1.8%)	0	0.018
Symptomatic SARS-CoV-2	10 (2%)	3 (0.5%)	0	0.035
Pneumonia	4 (0.7%)	0	0	0.05
Hospital admission	8 (1.5%)	0	0	0.012
Oxygen requirement	7 (1.3%)	0	0	0.006
ICU admission	2 (0.35%)	0	0	0.2
Death	2 (0.35%)	0	0	0.2

## Discussion

The current study highlights the influence of qualitative and quantitative humoral response monitoring early after full SARS-CoV-2 vaccination in predicting the risk of breakthrough SARS-CoV-2 infection in hematological patients. Patients lacking SCoV2-R-A at 3–6 weeks after vaccination had an increased risk of breakthrough SARS-CoV-2 infection. In addition, higher levels of SCoV2-R-A early after complete vaccination were linked to a lower risk of breakthrough SARS-CoV-2 infection and lower disease severity.

Hematological patients are historically characterized by a low humoral response rate with any vaccine-preventable disease [22, 23]. However, development of mRNA vaccines during the SARS-CoV-2 pandemic has overcome the poor serological response rates in this particular population (> 70%) [14, 19, 20, 24, 25]. The median 720.26 BAU/mL found in our cohort was similar to the results reported in other large series of patients with diverse hematological conditions (median values < 1 × 10^3^ BAU/mL) and significantly lower than those found in healthy individuals (> 1 × 10^3^ BAU/m) [26]. Although the clinical benefit of mounting a serological response is currently lacking in this scenario, to the best of our knowledge this is the first report providing evidence of its link with clinical efficacy. Notably, although prior SARS-CoV-2 infection was not significantly associated with lower risk of breakthrough SARS-CoV-2 infection in our series, none of these patients were infected after vaccination. We previously reported a higher rate of detectable SCoV2-R-A in this patient subset [21], whereas in the current study we were able to demonstrate higher SCoV2-R-A titers compared to SARS-CoV-2-naïve patients. It is likely that the higher antibody titers along with natural immunity may confer strong protection in these patients. The design of our registry (prospective longitudinal with several SCoV2-R-A determinations over time) enabled us to capture occurring breakthrough SARS-COV-2 infections through PCR screening in symptomatic patients or in asymptomatic patients before planned treatments but also by monitoring anti-N seroconversion in asymptomatic patients at different pre-specified time points.

SARS-CoV-2 infection in hematological patients mirrored national epidemiological data [1, 27]. The monitoring period during which our study was conducted (mostly from March 2021 to early December 2021) spanned the period between the fifth and sixth COVID-19 wave when the Delta variant was dominant in Spain. Thus, it is likely that our findings do not apply to the Omicron SARS-CoV-2 variant. In that period, the incidence of COVID-19 was relatively low [28]. This fact may explain in part the somewhat low incidence rate of SARS-CoV-2 infection (overall 6.39 per 100 persons-year) in the current study. Although hematological malignancies showed a higher risk of breakthrough infection compared to solid tumors [29], the rate observed in our cohort (2.6%) was similar to other series with comparable median follow-ups which included hematological patients (2.3%) [26] or health care workers (2.3%) [30]. However, we observed that the risk of breakthrough infection increased with longer follow-up. This fact could suggest either that a decrease in antibody titters over time may reduce protection (which formed the rationale behind boosters), or that the risk of being infected increases with longer exposure time in an ongoing pandemic.

The protective threshold levels of anti-SARS-CoV-2 antibodies below which the humoral defense against different SARS-CoV-2 variants is suboptimal have not yet been established. However, both binding and neutralizing antibodies are thought to be potential correlates of protection against COVID-19 and are correlated with each other [31, 32]. In fact, recent data in the general population suggest that higher levels of binding and neutralizing antibodies after the second dose correlate with a reduced risk of symptomatic infection [33, 34]. In line with these observations, our findings support that SCoV2-R-A titration early after vaccination could be an accurate strategy to predict breakthrough infection risk and could be useful in counseling additional vaccine doses or anti-SARS-CoV-2 monoclonal antibodies in this immunosuppressed population. This assumption is supported by the fact that patients without detectable antibodies had higher risk of breakthrough infection, patients with breakthrough infection had lower SCoV2-R-A titers and those with higher antibody titers showed lower disease severity.

A vaccine efficacy of 80% against symptomatic infection in the general population was achieved with 264 BAU/ml [33]. In the COVE trial, this threshold shows > 90% vaccine efficacy [34]. Based on these data, we empirically selected the cutoff of 250 BAU/mL to segregate our cohort into poor and good responders. The cutoff of 250 BAU/mL in our series predicted risk of breakthrough infection and disease severity. No patients with SCoV2-R-A > 250 BAU/mL were admitted to hospital due to SARS-CoV-2 in our cohort. Nevertheless, the kinetics wane of SCoV2-R-A waning after vaccination in this scenario remains to be determined in order to establish the best time points for serological monitoring and the optimal moment to administer further vaccine doses.

Finally, we observed an important reduction in COVID-19 severity, exemplified by reduced overall pneumonia (19%), symptomatic SARS-CoV-2 infection (48.6%) and mortality (8%) rates. Rates of these outcomes in hematological patients with COVID-19 during the first and second COVID-19 waves were as high as > 70%, > 90% and > 25%, respectively [1]. Our findings are in line with real world data, where both mRNA vaccines were shown to reduce symptomatic SARS-CoV-2 infection, COVID-19-related symptoms, hospital admissions and mortality in adults [35–38]. Although the severity of COVID-19 seems lower after vaccination, severely immunosuppressed patients still develop life-threatening disease. In these vulnerable patients, preventive transmission measures (hand washing, social distancing, wearing mask, etc.) are still highly recommended. However, the largest reduction in the incidence of respiratory virus infections in immunocompromised patients has been observed when preventive transmission measures have been instituted globally [39] as compared to when such measures are applied only to immunosuppressed patients and their caregivers; in fact, in these latter scenarios the time of onset and incidence of different respiratory virus infections (including SARS-CoV-2) in HSCT recipients strongly mirror those in the patients’ communities [1, 19].

The limitations of this study comprise the use of different serological tests, absence of neutralizing antibody testing, absence of cellular immune response analyses and the lack of molecular data regarding the SARS-CoV-2 variants in patients with breakthrough infections. However, most SARS-CoV-2 infections reported in our cohort occurred when the Delta variant was dominant in Spain. The performance of antibody titration with the omicron variant remains to be evaluated.

## Conclusion

We provide evidence that serological monitoring after SARS-CoV-2 vaccination could be useful in identifying hematological patients at high risk of breakthrough SARS-CoV-2 infection. SCoV2-R-A levels link with protection in this vulnerable population being 250 BAU/mL a potentially discriminative cutoff for non-Omicron SARS-CoV-2 variants. Finally, severity of SARS-CoV-2 infection in hematological patients has experienced an encouraging improvement in the post-vaccine period.

## Supplementary Information

**Additional file 1: Table S1.** Characteristics of serological assays used in the study. **Table S2.** Commercial PCR test available in participating centers.

## Data Availability

Data are available upon request by email to the Spanish hematopoietic transplant and cell therapy group (GETH-TC).
