# Supplementary material for: SARS-CoV-2 vaccine response and rate of breakthrough infection in patients with hematological disorders
Source: J Hematol Oncol. 2022 May 7;15:54. doi: 10.1186/s13045-022-01275-7 (PMC9077637; doi:10.1186/s13045-022-01275-7)
Supplement: Supplementary file 1 — Additional file 1: Table S1. Characteristics of serological assays used in the study. Table S2. Commercial PCR test available in participating centers. [file 13045_2022_1275_MOESM1_ESM.docx]

**Table S1.** Characteristics of serological assays used in the study.

| Test | Manufacturer | Antibody target | SARS-CoV-2 antigen | BAU/mL |
| --- | --- | --- | --- | --- |
| - Architect SARS-CoV-2 IgG Quant II chemiluminescent microparticle immunoassay | (Abbott Diagnostics, Ill, USA) | IgG | RBD | AU x 0.142 |
| - Abbott ARCHITECT SARS-CoV-2 IgG | (Abbott Diagnostics, Ill, USA) | IgG | N |  |
| - Liaison SARS-CoV-2 S1/S2 IgG chemiluminescent assay | (DiaSorin S.p.A., Saluggia, Italy) | IgG | S1/S2 | AU x 2,6 |
| - MAGLUMI 2019-nCoV IgG chemiluminescent assay | (SNIBE—Shenzhen New Industries Biomedical Engineering Co., Ltd., Shenzhen, China) | IgG | S and N | AU/4.33 |
| - Elecsys anti-SARS-CoV-2 S | Roche Diagnostics (Pleasanton, CA, USA) | Total antibody (IgG, IgM, IgA) | RBD | U x 0.98 |
| - Elecsys® Anti-SARS-CoV-2 N | Roche Diagnostics (Pleasanton, CA, USA) | IgG | N |  |
| - Atellica SARS-CoV-2 | Siemmens <https://www.siemens-healthineers> (Germany) | Total IgG,IgM, IgA | RBD | U x 21.8 |
| S, Spike protein; N, nucleocapsid protein; RBD, receptor binding domain; AU, arbitrary units; U, units | | | |  |

**Table S2.** Commercial PCR test available in participating centers

| Alinity m SARS-CoV-2 Assay - Abbott Molecular (Ill, USA) |
| --- |
| Abbott RealTime SARS-CoV-2 Assay (Abbott (Ill, USA) |
| LightMix® Modular SARS-CoV (COVID-19) (Roche Diagnostics, Pleasanton, USA) |
| Aptima® SARS-CoV-2 Assay (Panther® System) (Hologic, Marlborough, Massachusetts, USA |
| TaqPath COVID-19 CE-IVD RT-PCR (Thermofisher Scientific Waltham, Massachusetts, USA |
| SARS-CoV-2 RT-PCR Vitro (Sevilla, Spain) |
| Xpert® Xpress SARS-CoV-2 Sunnyvale, California, USA |
| SARS-CoV-2 REAL TIME PCR KIT (Vircell, Granada, Spain) |
